# Supplementary figures and images for: A common cis-regulatory variant impacts normal-range and disease-associated human facial shape through regulation of PKDCC during chondrogenesis
Source: eLife. 2024 Mar 14;13:e82564. doi: 10.7554/eLife.82564 (PMC10939500; doi:10.7554/eLife.82564)

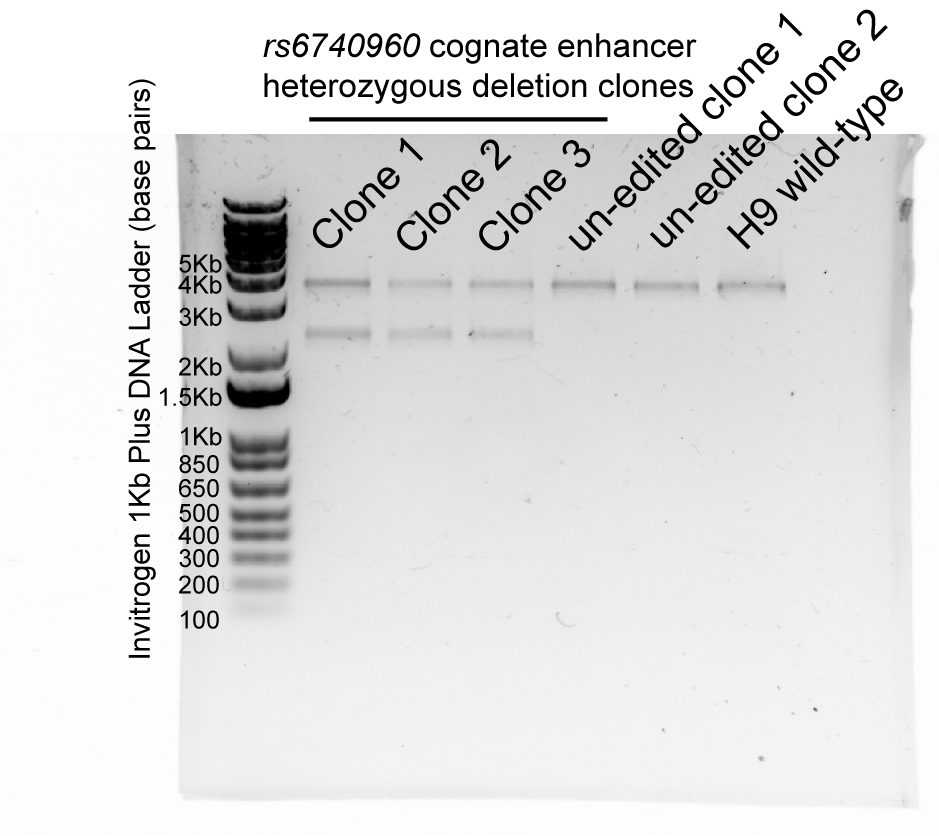

Supplement: Figure 3—figure supplement 3—source data 1. — SCN file can be viewed using Bio-Rad’s Image Lab Software. [file elife-82564-fig3-figsupp3-data1.zip › Figure_3_Figure_Supplement_3_source_data/Figure3_FigureSupplement3_Gel_LABELEDFILE.tif]
